# Climate change impacts detection in dry forested ecosystem as indicated by vegetation cover change in —Laikipia, of Kenya

**DOI:** 10.1007/s10661-018-6630-6

**Published:** 2018-03-29

**Authors:** Kiambi Gilbert M’mboroki, Shem Wandiga, Silas Odongo Oriaso

**Affiliations:** 1 State Department of Livestock, Ministry of Agriculture, Livestock Development and Fisheries, Nairobi, Kenya; 2 Institute of Climate Change and Adaptation, University Nairobi, Nairobi, Kenya

**Keywords:** Detection, Climate change adaptations, Impacts, Dry forested ecosystem, Land use, Vegetation cover, Yaaku

## Abstract

The objective of the study was to detect and identify land cover changes in Laikipia County of Kenya that have occurred during the last three decades. The land use types of study area are six, of which three are the main and the other three are the minor. The main three, forest, shrub or bush land and grassland, changed during the period, of which grasslands reduced by 5864 ha (40%), forest by 3071 ha (24%) and shrub and bush land increased by 8912 ha (43%). The other three minor land use types were bare land which had reduced by 238 ha (45%), river bed vegetation increased by 209 ha (72%) and agriculture increased by 52 ha (600%) over the period decades. Differences in spatiotemporal variations of vegetation could be largely attributed to the effects of climate factors, anthropogenic activities and their interactions. Precipitation and temperature have been demonstrated to be the key climate factors for plant growth and vegetation development where rainfall decreased by 200 mm and temperatures increased by 1.5 °C over the period. Also, the opinion of the community on the change of land use and management was attributed to climate change and also adaptation strategies applied by the community over time. For example unlike the common understanding that forest resources utilisation increases with increasing human population, Mukogodo dry forested ecosystem case is different in that the majority of the respondents (78.9%) reported that the forest resource use was more in that period than now and also a similar majority (74.2%) had the same opinion that forest resource utilisation was low compared to last 30 years. In Yaaku community, change impacts were evidenced and thus mitigation measures suggested to address the impacts which included the following: controlled bush management and indigenous grass reseeding programme were advocated to restore original grasslands, and agricultural (crop farming) activities are carried out in designated areas outside the forest conservation areas (ecosystem zoning) all in consultation with government (political class), community and other stakeholders. Groups are organised (environmental management committee) to address conservation, political and vulnerability issues in the pastoral dry forested ecosystem which will sustain pastoralism in the ecosystem.

## Introduction

An understanding of land use or land cover change at local, regional and global scales is important in an increasingly human dominated biosphere. The terms land cover and land use, although often used interchangeably, their actual meanings are quite distinct. Land cover refers to the surface cover on the ground, while land use refers to the purpose the land serves. In this study, the terms are used interchangeably because both aspects are in consideration. Change detection is an important process in monitoring and managing natural resources, land use change analysis, monitoring of shifting cultivation and assessment of deforestation because it provides quantitative analysis of the spatial distribution of the population of interest.

Laikipia County is predominantly a livestock rearing county with ranches occupying over 50% of the entire land and 580 km^2^ of land covered by six gazetted and one non-gazetted forest. These forests are either natural or artificial in establishment, for instance Mukogodo dry forest is one of natural forests not gazetted at the lee ward side of Mount Kenya and inhabited by an indigenous and minority community known as the Yaaku. However, some factors such as forest fires, deforestation and grazing have largely led to depletion of the forest cover over the years. Poles, pastures, wood fuel and timber are the forest main products. The provision of setting of bee hives, research ground on flora, natural herbs and wildlife habitat for instance for elephants and birds has all been provided by this forest. In Mukogodo, ecotourism is a dominant activity through conservation of natural forests particularly at Iligwesi, kurikuri and lekuruki community conservancies. .

The other land use patterns in the county are pastoralism, mixed farming, ranching, agro-pastoral, marginal mixed farming, much influenced by the climatic conditions and the ecological zones.

The Yaaku community resides in the group ranches of kurikuri and lekuruki which include the forested ecosystem of Mukogodo.

Laikipia County Government (LCG) (2013–2017) indicates that most vulnerable areas to climate change phenomenon are forested dry lands of Kenya. This is brought about by the friable nature of the environment that has been caused by encroachment by agricultural activities linked to increased human population and followed by unsustainable land use activities. In this regard, the frequency and severity of both droughts and floods are already high and are expected to increase in coming years (LCG, 2013–2017). Availability of rainfall determines the smallholder farming and livestock production though the latter is dominant in the Mukogodo ecosystem. Major impacts of droughts on smallholder activities have increased food insecurity (food shortage and poverty) and loss of livelihoods (LCG, 2013–2017).

Pastoral communities in Laikipia have similar climate change impacts and concerns with other forested dry lands of Kenya, although, means to food security of communities varied from place to place as do the adaptation strategies to environmental hazards such as drought and floods, of which Laikipia County is no exception. Therefore, each agro-ecological zone has distinct challenges in maintaining food security, which often cut across all the sectors. Thus, the forested dry land of Laikipia County has different and distinct challenges from other agro-ecological zones.

### Study area

The study was conducted in Mukogodo East Ward, which is in Laikipia North Sub-county of Laikipia County of Kenya. The Laikipia County lies between latitude 0° 18″ and 0° 51″ north and between longitude 36° 11″ and 37° 24′ east. It occupies an area of 9462 km^2^ (2,338,111 acres) (LCG, 2013–2017).

**Fig. 1:**
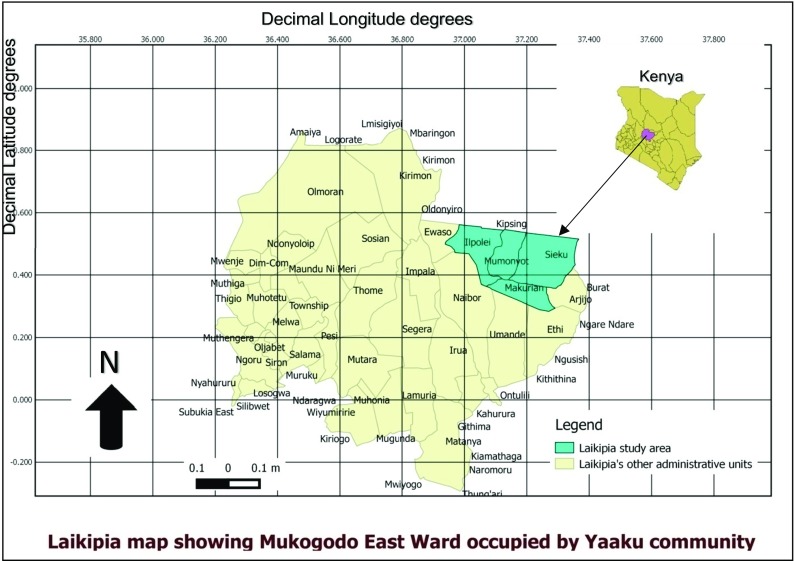
A map of Laikipia County showing the study site

As indicated in the map of the study area, Fig. 1, the Mukogodo East ward is composed of four locations, namely Makurian, Sieku, Mukogodo (Mumonyot) and Iipolei locations: Mukogodo East Ward borders, Mukogodo west to the west, Umande Ward to the south, Meru County to the east and Isiolo County to the north. The area is mainly dry forested ecosystem with grass undergrowth. The study site and the hazard (climate change) were identified by community participatory methodology known as community-managed disaster and risk reduction (CMDRR).

The pastoralist groups are found on the arid and semi-arid lands (ASALs), which constitute 84% of the country’s area unlike the hunters and gatherers whose ecosystem is not well defined (IFAD 2012). As reported by IFAD (2012), the difference between hunter, gatherers and pastoralists is not clear-cut since some hunters and gatherers keep cattle and many pastoralists mix livestock herding with other subsistence strategies (cultivation, hunting, gathering). The Yaaku is in this category where they are among the old Cushite groups in the Rift Valley of Tanzania and Kenya that became associated with various Nilotic tribes like clients, mostly as self-defence for their own preservation under the various waves of Nilotic migration into their ancestral area (Orville 1996).

The Yaaku are associated with the Maasai of Kenya and majority speak Maa language and are referred to as the *Mukogodo* Maasai. *Athii* and *Mwoko*re are names given to the Yaaku in the traditions by highland Bantus like Kikuyu and Meru. The Maasai called these early Cushitic people by the name Dorobo. The *Dorobo* are not one tribe but referred to various original forest-dwelling hunters-gatherers (Southern or Eastern Cushite). One connotation of *Dorobo* is poor people who do not own cattle (Orville 1996). The Yaaku consider themselves Maasai and speak Maa language because they have gotten completely into Maasai culture and language. Between 1925 and 1936, they left their Cushitic language Yaaku for the Eastern Nilotic Maasai language (Yaaku dice database—Internet). The hunters and gatherers have knowledge of the forests natural resources, its animals and its trees, the individual properties and use of thousands of plants, where to find and gather honey and how to use them in a sustainable way; this has not only sustained the hunters and gatherers themselves but has benefited their neighbours, with whom exchange networks have been established and functioned for centuries. Their neighbours are the Maasai and Samburu who are pastoralists and the Meru who are agro-pastoralists.

### Topography

At Ewaso Nyiro Basin in the north, the county altitude lies between 1500 m above sea level and 2611 m in the south. Marmanet Forest is found at a height of 2611 m above sea level. Mukogodo and Loldaiga forests are other areas of high altitude in the eastern part of the County. These areas lie at 2200 m above sea level (LCG, 2013–2017). As illustrated in Figure 1, the Mukogodo Forest is the home of Yaaku community who occupy Mukogodo and Sieku locations of Mukogodo East Ward of Laikipia North Sub County of Laikipia County. Figure 1 shows the Mukogodo East Ward which is the home of Yaaku community.

### Population

According to the 2009 Kenya National Bureau of Statistics (KNBS), the total population for the Laikipia stands at 399,227 people; of these, 200,602 are females and 198,625 males. The population projection for year 2017 is 479,072 persons. The study targeted a small pastoralist community of about 4000 inhabitants “not certain” for they were included in category of “others” in 2009 population census (IFAD 2012), called the Yaaku living in Mukogodo and Sieku locations of Mukogodo East Ward of Laikipia County.

### Vegetation cover

Vegetation is an assemblage of plant species of the ground cover and canopy they provide. In Mukogodo dry forested ecosystem, the ground cover is mainly grasses and open canopy made of Cedar, Podo, Olive, Croton, Lichen, Ferns and Orchids. The shrubby vegetation mainly is Euclear and Tachonathus. The ecosystem is determined by the bioclimatic zone between the Mount Kenya wet forest and the north eastern savannahs of Kenya. Therefore, this vegetation cover is used as an indicator to evaluate terrestrial environmental conditions. Changes in the spatiotemporal patterns of vegetation alter the structures and functions of the landscape, thereby affecting ecological processes.

### Land use and land cover

Whereas land cover indicates the physical land type such as forest or open water, consequently, land use documents how people are using the land and therefore land use data documents show how much of a region is covered by forests, wetlands, agriculture, bare areas and water types. An understanding of land use/land cover change at local, regional and global scales is important in an increasingly human-dominated biosphere. The terms land cover and land use, although often used interchangeably, their actual meanings are quite distinct. Land cover refers to the surface cover on the ground, while land use refers to the purpose the land serves (Shiraz 2014). Land cover/land use has been used extensively to derive a number of biophysical variables, such as vegetation index and biomass (Shunlin 2008). Land use and land cover are important components in understanding the interactions of the human activities with the environment, and thus, it is necessary to be able to simulate changes (Kuldeep and Kamlesh 2011). Land use and land cover change have become a central component in current strategies for managing natural resources and monitoring environmental change (Kuldeep and Kamlesh 2011). The properties measured with remote sensing techniques relate to land cover, from which land use can be inferred, particularly with supplementary data or a priori knowledge (Noam 1999). The detection of land use/land cover change is the process of identifying differences in the state of an object or phenomenon by observing it at different times (Singh 1989).

The Mukogodo dry forested ecosystem has various land uses over the period of investigations ranging from gathering of fruits, honey harvesting, medicinal plants harvesting grazing, agriculture by the inhabitants the Yaaku community and tourism.

### Change detection

Change detection for GIS (geographical information systems) is a process that measures how the attributes of a particular area have changed between two or more time periods. Therefore, change detection involves comparing aerial photographs or satellite imagery of the area taken at different times or epoch. Change detection is an important process in monitoring and managing natural resources, land use change analysis, monitoring of shifting cultivation and assessment of deforestation because it provides quantitative analysis of the spatial distribution of the population of interest. The increasing impact of land cover change on the environment has been an issue of concern in the developed and the developing countries with consequential effects on sustainable development and long-term impact on the agricultural and other sectors of the economy. This involves the spatial and non-spatial data to query new information. Vegetation cover analysis has been carried out in Kenya by Tracy et al. (2005) and Ayuyo and Sweta (2014) all of whom carried land change detection for Mau Water tower.

### Climate change adaptations

Climate change adaptation is a response to global warming and climate change, which seeks to reduce the vulnerability of social and biological systems to relatively sudden change and thus offset the effects of global warming. Adaptation is especially important in developing countries since those countries are predicted to bear the brunt of the effects of global warming. That is, the capacity and potential for humans to adapt (called adaptive capacity) are unevenly distributed across different regions and populations, and developing countries generally have less capacity to adapt.

### Dry forested ecosystem

Temperatures are high all year, but there is a better-developed dry season than in the tropical rain forest. Evapotranspiration exceeds precipitation for enough of the year to have a significant effect on the vegetation. Edaphic conditions (dryer, better-drained soil) may produce this vegetation type in the rain-forest zone. Soils are essentially like those of tropical rain forests, with the same processes. The deciduousness of most tree species is a significant difference from the tropical rain forest. Many evergreen tree species of the rain forest become deciduous in this zone. Growing conditions are not so optimal; thus, the tree canopy is lower (10–30 m) than in the tropical rain forest and the trees less dense where drought is more extreme. The undergrowth is often dense and tangled because of greater light penetration. Species diversity is invariably lower than in nearby tropical rain forests. Environmental stress increases with instability (seasonality) of the environment, and fewer plants and animals can generate homeostatic mechanisms (for internal stability) to cope. Trees have thicker bark (anti-fire adaptation), thicker and smaller leaves (anti-desiccation adaptation), thorns (anti- herbivore adaptation), longer roots (to reach deeper water table) and other features along a gradient toward the well-developed drought adaptations of woody plants of the savanna and desert zones, The high productivity during the rainy season, coupled with relief from rains during the dry season, makes this a favourable environment for humans and domestic stock; so much of the zone has been cleared and developed for pastureland as well as agriculture.

Mukogodo forest, spanning 30,000 ha of rugged hillside terrain in North West Laikipia, is the largest and best preserved of all the region’s forests. That it has remained almost wholly intact is a tribute, not only to a relatively remote location and dry surroundings, but also to the astute custodianship of the Yaaku hunter-gatherers who, for centuries, have been living in the forest (CGL ( 2017). The forest and the adjacent areas (community conservancies) totals to about 50,000 ha which were under study.

This community has succeeded, in the absence of conservation interventions from without, in warding off loggers intent on plundering the area’s Pencil Cedar trees, and in regulating access by pastoralist communities to grazing in the forest during dry spells. The integrity of the forest has been widely respected as a result. And today, Mukogodo is extolled as a model of sustainable use, and of the capacity of local people to safeguard the forest resources on which they depend.

As well as impressive stands of Pencil Cedar and Olive, Mukogodo boasts some magnificent Crotons, *C. megalocarpus*, tall, graceful trees that are typical of dry forest habitats, having flattish, spreading crowns and layered silvery leaves. The nectar of the creamy-white Croton flowers attracts countless bees, providing local community groups with a rich source of honey. In the northern reaches of the forest, other plants—such as giant *Euphorbia candelabrum* trees and “blue-bark” Commiphoras, *C. baluensis*—come into their own. Before the rains, the Dombeya, *D. rotundifolia*, produces exquisite bunches of pale pink flowers resembling cherry blossoms.

## Methods

The study population composed of 800 households in Mukogodo and Sieku locations in Mukogodo East Ward while the sampling frame, from which the study sample was drawn constituted all the households living in the nine villages of these two locations. The unit of analysis was the household, and the subject of analysis (the respondent) was the head of the household or their representative.

In each of the nine villages, a list of the households was compiled during the process of community managed disaster and risk reduction (CMDRR) as used by Abdi and Cord Aid (2011), IIRR and Cord Aid (2013) and CARE International (2015), and systematic sampling was used to pick numbers of households (actually about 30% of households) from each village (Borg and Gall 2003). Then, random sampling was undertaken among the systematically selected households in each village to constitute a study sample of 240 households.

Two formulae (from Mugenda and Mugenda 1999 and Kathuri and Pals 1993) were used for computing the study sample size but yielded rather large sample sizes that could not be sustained by the available resources for the study. According to Kathuri and Pals (1993), a minimum of 100 is recommended for a survey research and gives a reasonable unit for analysis.

Borg and Gall (2003) indicated that at least 30% of the total population is representative. Thus, 30% of the accessible population is enough for the sample size. Thus, in this study, 30% of 800 households were 240 respondents.

Hence, resorting to the provisions of the Statistical Package for the Social Sciences (SPSS) programme suggests that any sample size of 200 and above will allow perfect functioning of all the analytical procedures provided by the programme.

A socio-ecological survey using a structured questionnaire was used to collect respondent’s opinion on climate changes and adaptation on land use and management in specific based on livelihood capitals(natural capital) for the last three decades from 1986 to 2015. Data from social ecological survey was analysed after entry in to Statistical Package for Social Sciences (SPSS) to get the respondents’ views of land use and management within the three decades. The data on average annual rainfall and daily maximum and minimum temperatures from 1986 to 2015 was collected from Laikipia Meteorological Station in Kalalu of Mukogodo East Ward (Laikipia North) which is within the study area.

The remote-sensed data from Landsat images of the area for last three decades was collected from regional centre for mapping of resources for development (RCMRD) to determine land use changes. In the classification, maps were generated using specialist software (IMPACT Tool, Erdas and ArcGIS). The maps showed the land cover status of the four epochs, 1984, 1995, 2004 and 2014, of which changes between each of the epoch for 1984–1995, 1995–2004 and 2004–2014 gave the statistics of change. Field validation was done to improve the classification and to come up with class cover validation and errors of classification computed.

The area was delimited by the Mukodogo East Ward within Laikipia County and which enclosed the Mukodogo Forest which was the home of the Yaaku community. Landsat imageries from regional centre for mapping of resources for development (RCMRD) were analysed by use of remote sensing computer software Erdas imagine (2014) and classified images were input into maps composition by ArGIS to give the land use trends as from 1984 to 2014. The study site was visualised by Google Earth for comparison with present situation as form of ground check, accompanied by ground visits to some of the areas. The changes were obtained by classifying Land sat images for four epochs: 1984, 1995, 2004 and 2014.

### Availability of data and materials

The data is deposited at the University of Nairobi because this article is part of bigger thesis report presented to the university but not published.

## Results

**Plate 1:**
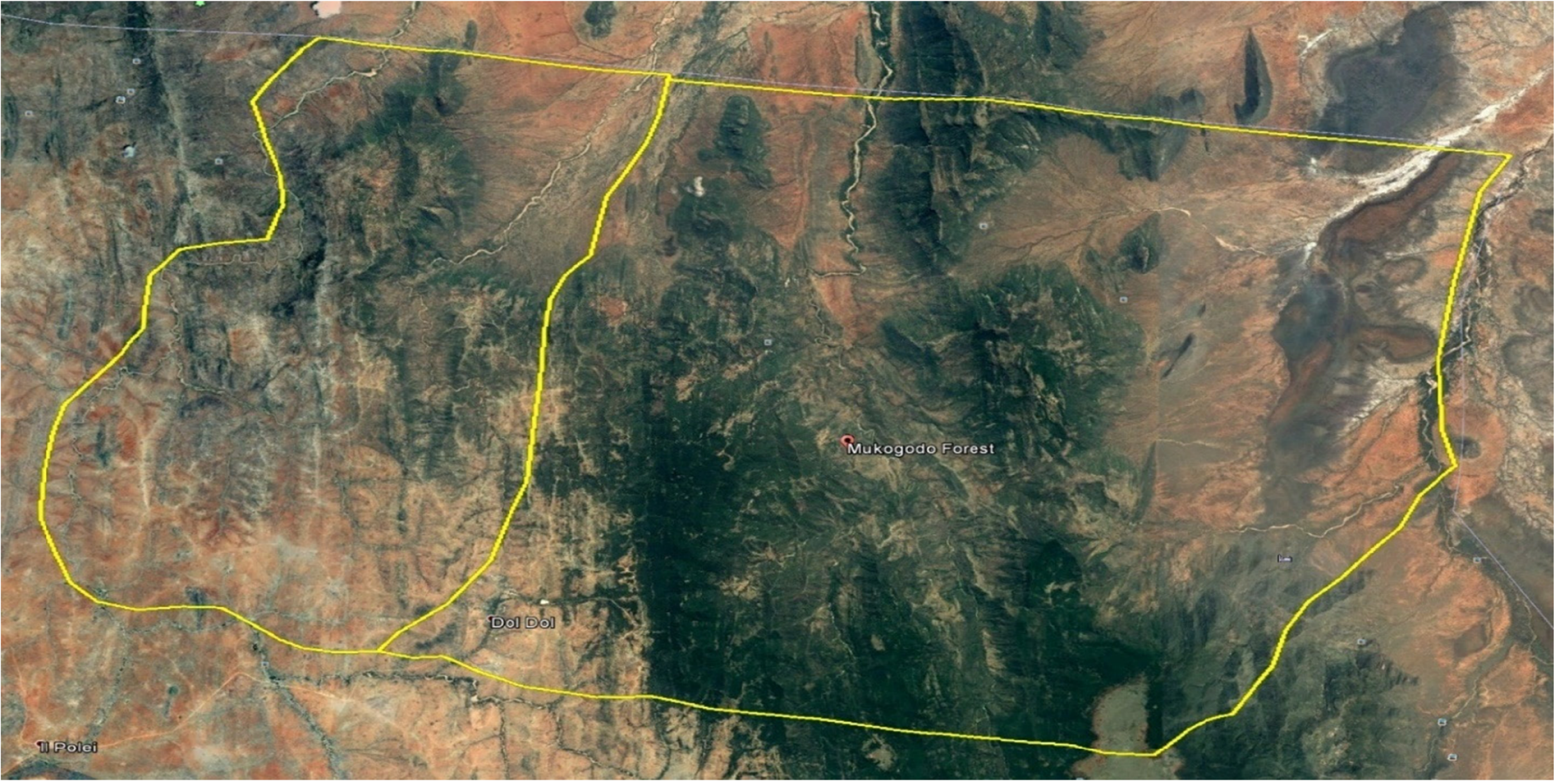
Mukogodo East Ward and part of the enclosed Mukogodo forest

The study site was visualised by Google Earth for comparison with present situation as form of confirmation by ground visits to some of the areas. Plate 1 shows Google Earth visualisation of Mukogodo East Ward and part of enclosed Mukogodo forest.

**Plate 2:**
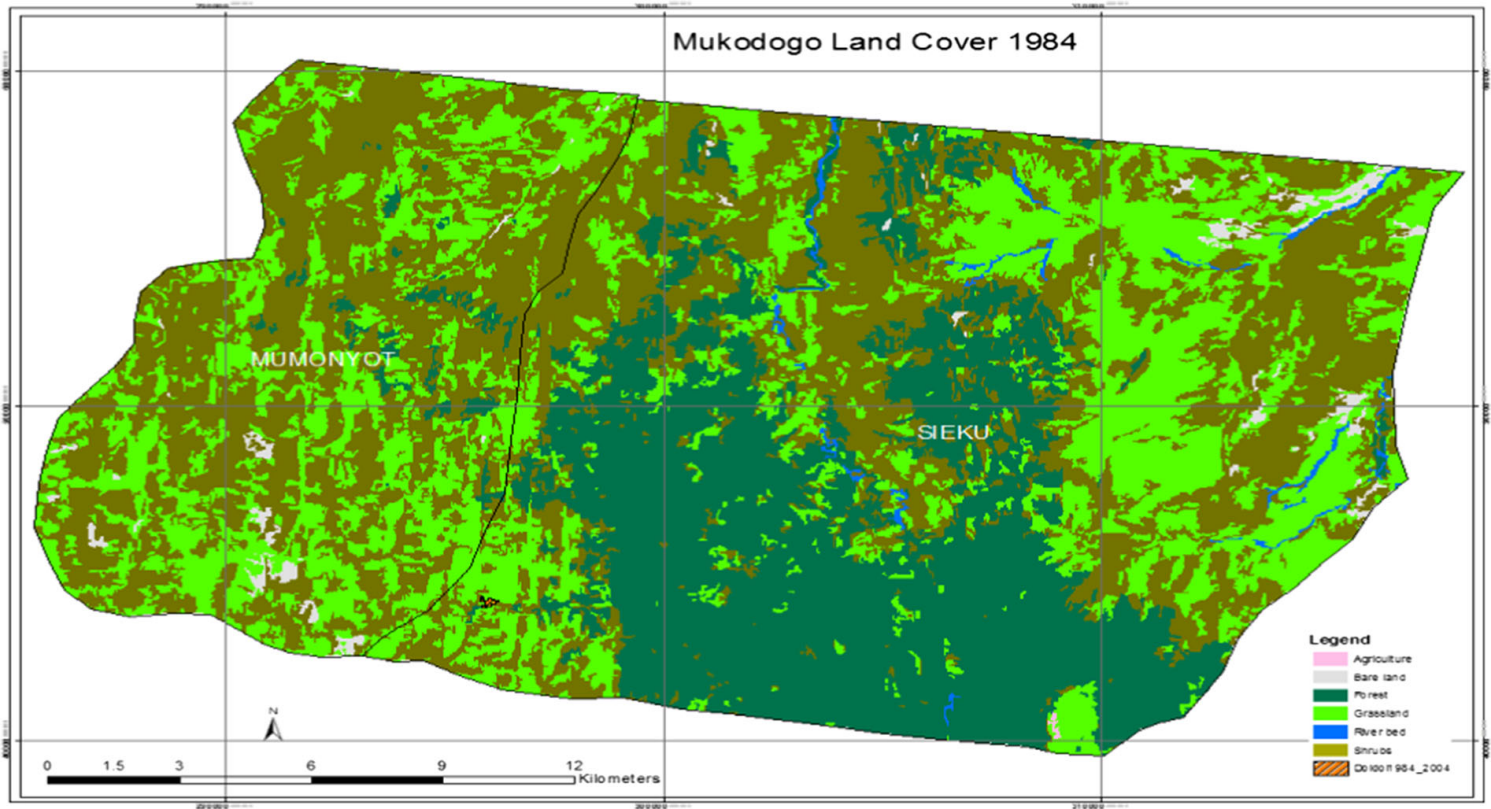
Mukogodo land cover 1984

As was visualised from the Google Earth image (Plate 1), there were a lot of spaces within the forest ecosystem and most of those spaces were earlier covered by grassland and in the recent past (notably seen in the image of 2014 Plate 4), these areas are now covered by shrubs and woodland that had little value to pastoralist. Additional information, for example the growth of Doldol town, was extracted from Google Earth image. Doldol town was seen to expand from 7 to 14 ha between 1984 and 2014. There were other small settlements, but they were too small to be mapped at this scale. Plate 2 shows Mukogodo land cover in 1984.

As indicated in Plate 2, the epoch of 1984 had very little agricultural activity and the area was covered by forest (the green and dark green colour in Plate 2). This epoch is used by the study as a base map in getting the land use changes in the last three decades.

**Plate 3:**
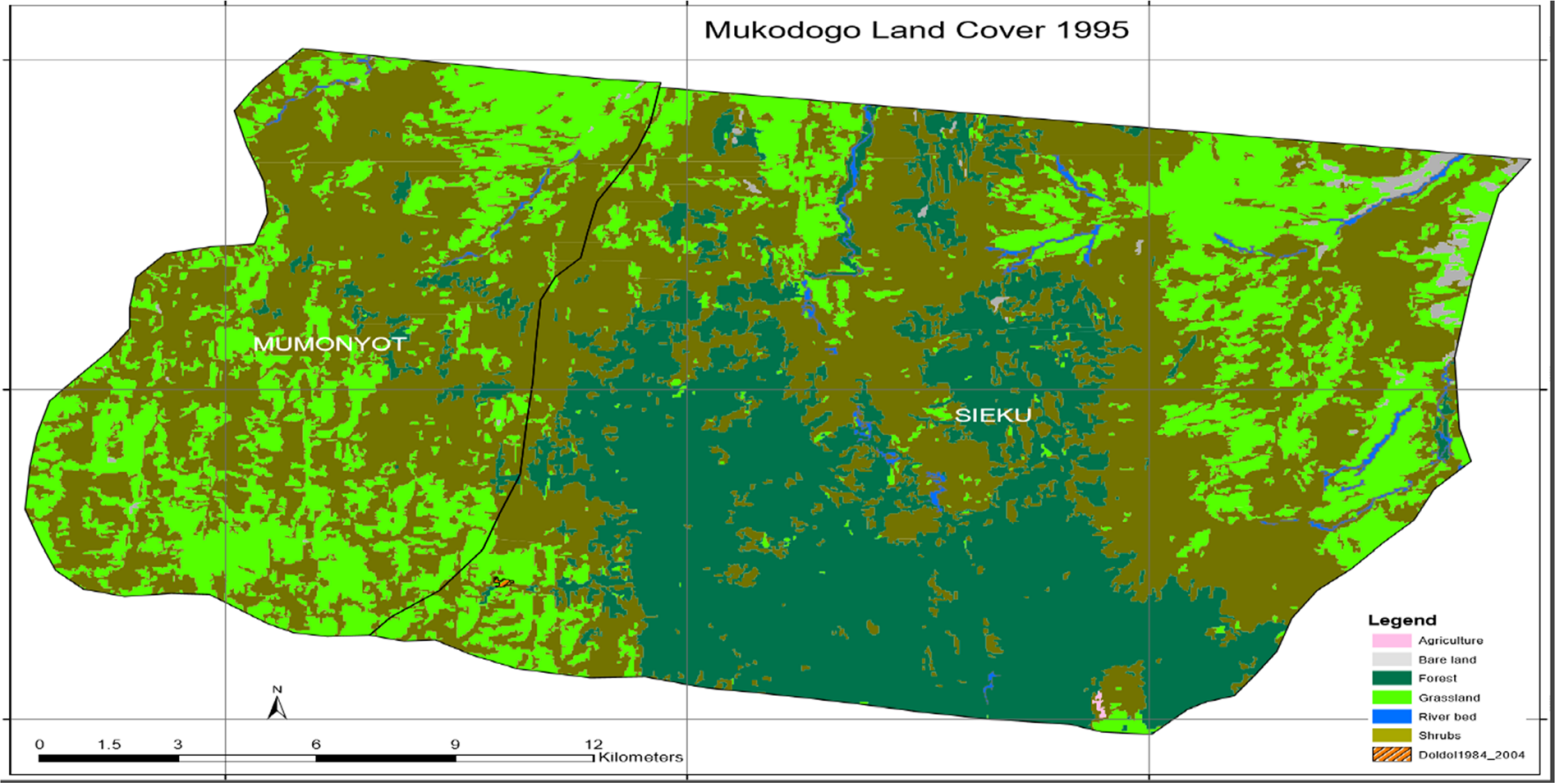
Mukogodo land cover 1995

Plate 3 shows Mukogodo land cover 1995.

**Plate 4:**
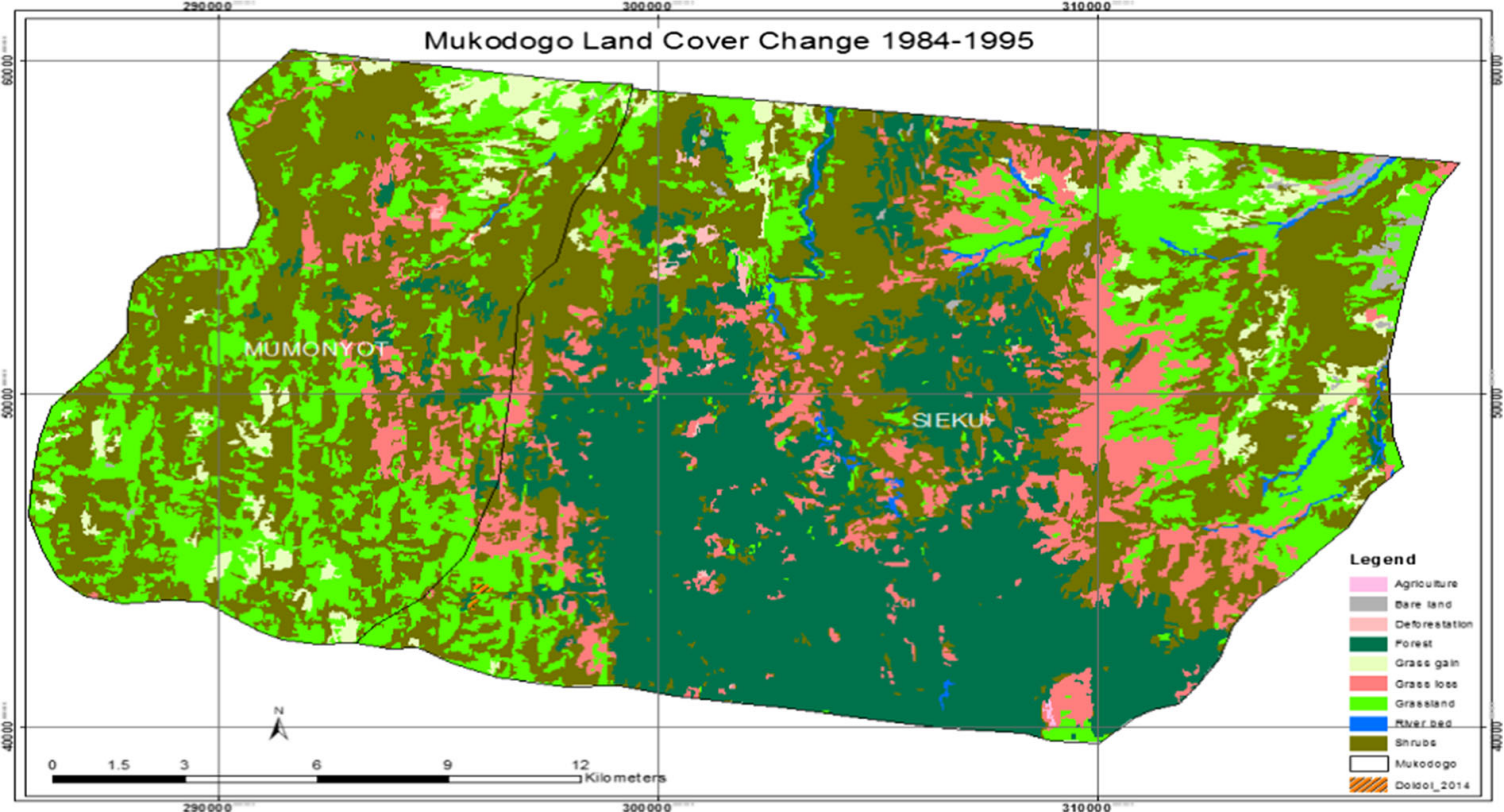
1984 to 1995 change map

As indicated in Plate 3, the epoch of 1995 had very little agricultural activity and the area was covered by forest (the green and dark green colour in Plate 3). In this epoch, shrub land had been noticed encroaching areas which had grassland. Plate 4 shows Mukogodo land cover change 1984 to 1995 (overlays map).

As indicated in Plate 4, the period 1984 to 1995 the grasslands had decreased but agriculture land remained unchanged. However, there was regeneration of grass in some area while there was loss in others. In general, there was net loss of grassland and forest was deforested. Table 1 summarises land use change in 1984 to 1995 as indicated in Plates 2, 3 and 4.

**Table 1:** 1984–1995 epochs

Land_cover_change1984_1994mod							
FID	Shape	ID	Tl_class_1	1984_LC	1995_LC	Change	Area
0	Polygon	14	14	Grassland	Grassland	Grassland	9887.578861
1	Polygon	40	12	Shrubs	Shrubs	Shrubs	19,297.830684
2	Polygon	147	11	Bare land	Bare land	Bare land	238.417348
3	Polygon	220	14	Shrubs	Grassland	Grass gain	1731.422213
4	Polygon	898	9	Forest	Forest	Forest	12,684.397535
5	Polygon	108	14	River bed	River bed	River bed	299.99352
6	Polygon	117	14	Grassland	Shrubs	Grass loss	4835.685697
7	Polygon	182	9	Forest	Shrubs	Deforestation	127.53
8	Polygon	125	12	Agriculture	Agriculture	Agriculture	9.36

**Plate 5:**
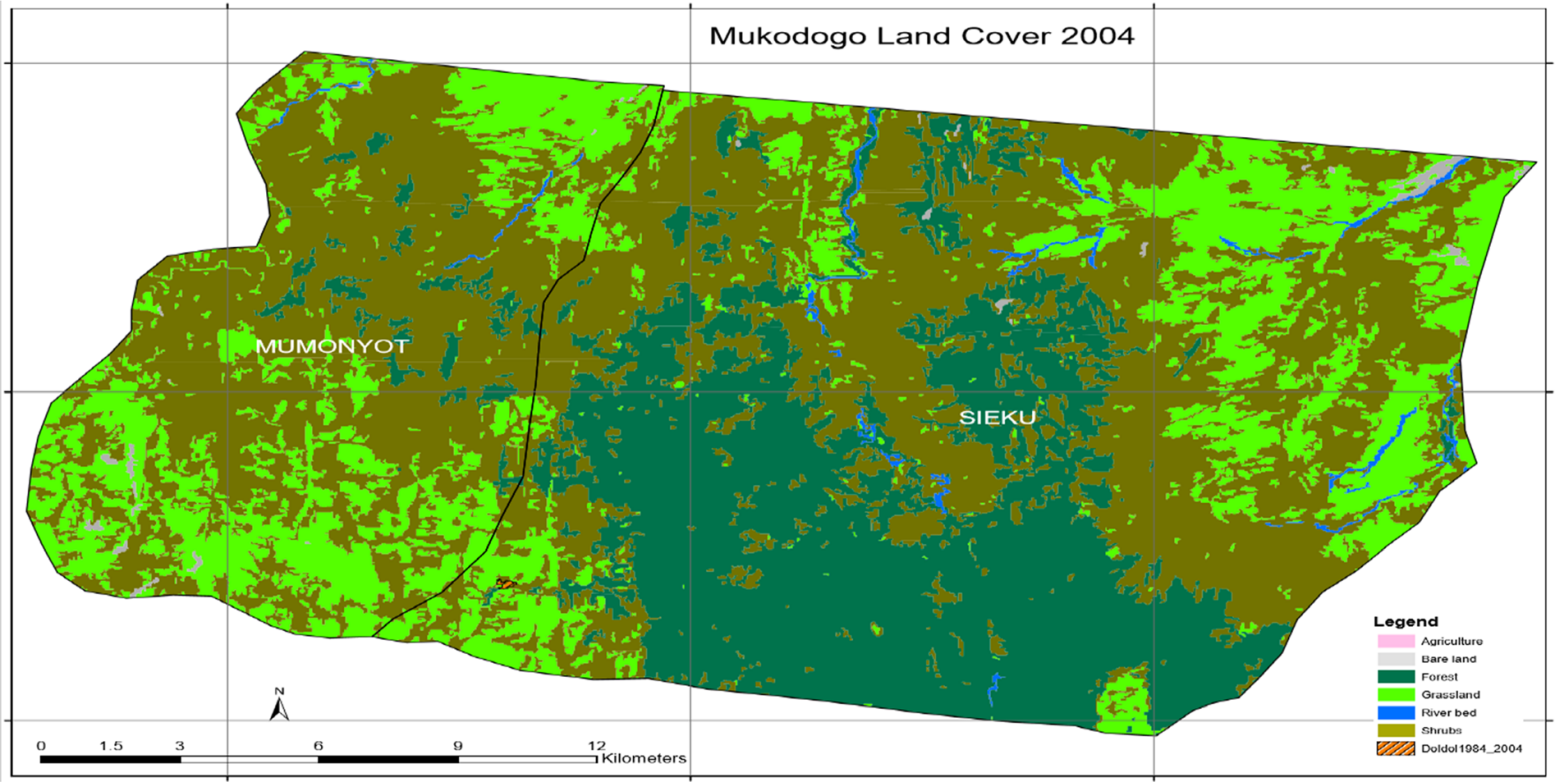
Mukogodo land cover 2004

As indicated in Table 1, between 1984 and 1995, 9888 ha of grassland and 9 ha of agriculture land remained unchanged. However, there was a regeneration of 1731 ha of grassland in some areas and a loss of 4836 ha in others. Therefore, in total, there was a loss of 3105 ha of grassland. In the same period, 127 ha of forest was deforested. Plate 5 shows Mukogodo land cover 2004.

**Plate 6:**
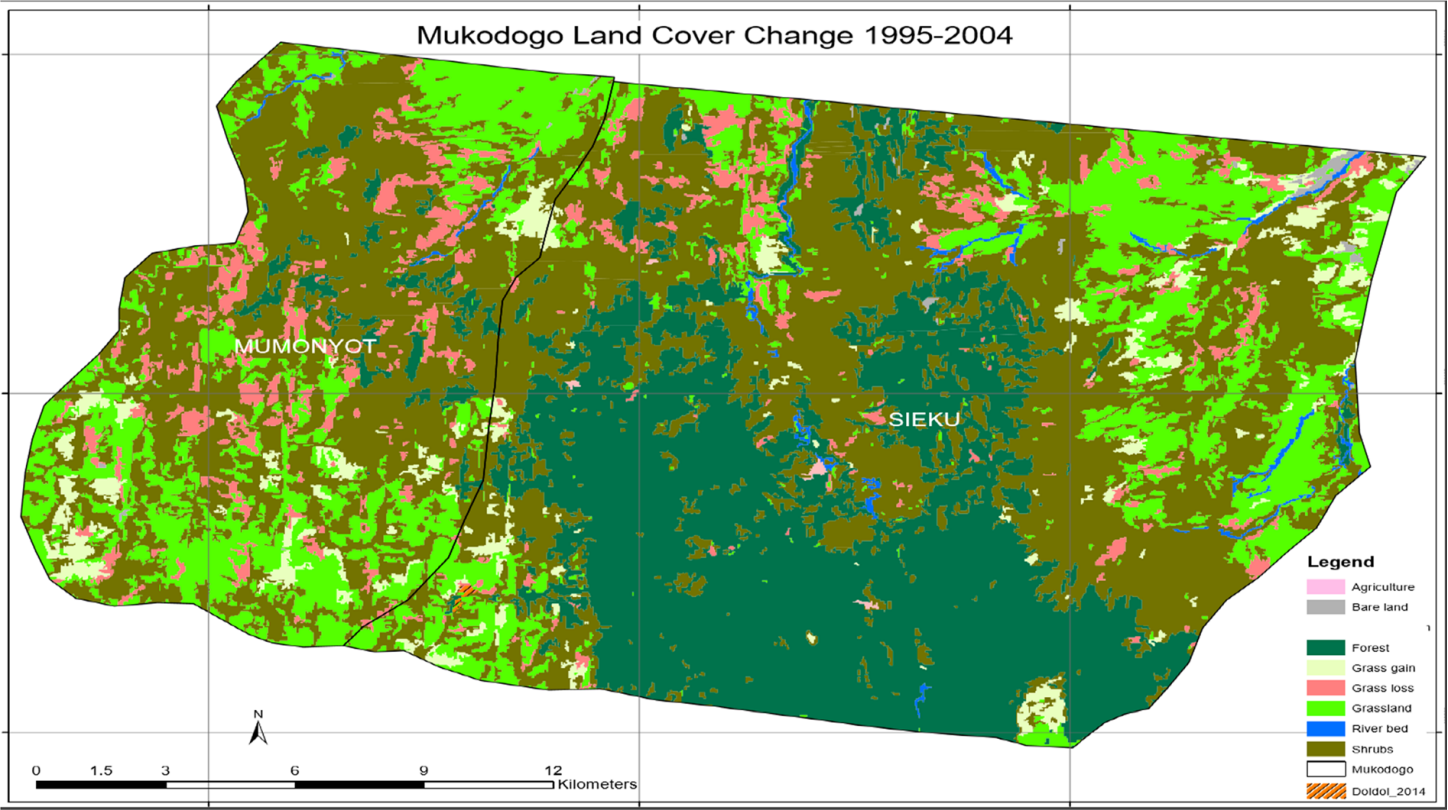
1995 to 2004 change map

As indicated in Plate 5, the epoch of 2004 had no agricultural activity at all and the area was covered by forest (the green and dark green colour in Plate 5). In this epoch, shrub land had been noticed encroaching areas which had grassland which was reducing gradually. In deep discussion in focus group, it came out that the drought of 2000 to 2001 had discouraged the few farming households in Mukogodo area and stayed without farming up to 2005. Plate 6 shows Mukogodo land cover change 1995 to 2004 (overlays map).

As indicated in Plate 6, in the period 1995 to 2004, the grasslands were lost and forested areas remained unchanged. Some areas of grassland regenerated while others lost grass cover giving a net grass loss. Deforestation continued and no evidence of agricultural activity in this epoch. Table 2 summarises the land use change in 1995 to 2004 as indicated in Plates 5 and 6.

**Table 2:** 1995–2004 epochs

T1_class_1	1995_LC	2004_LC	Change	Area
14	Grassland	Grassland	Grassland	9213.474869
12	Shrubs	Shrubs	Shrubs	22,507.795209
11	Bare land	Bare land	Bare land	122.545048
14	River bed	River bed	River bed	330.50352
14	Grassland	Shrubs	Grass loss	2405.526205
9	Forest	Forest	Forest	12,919.207535
14	Shrubs	Grassland	Grass gain	1583.733473
14	Forest	Shrubs	Deforestation	29.43

**Plate 7:**
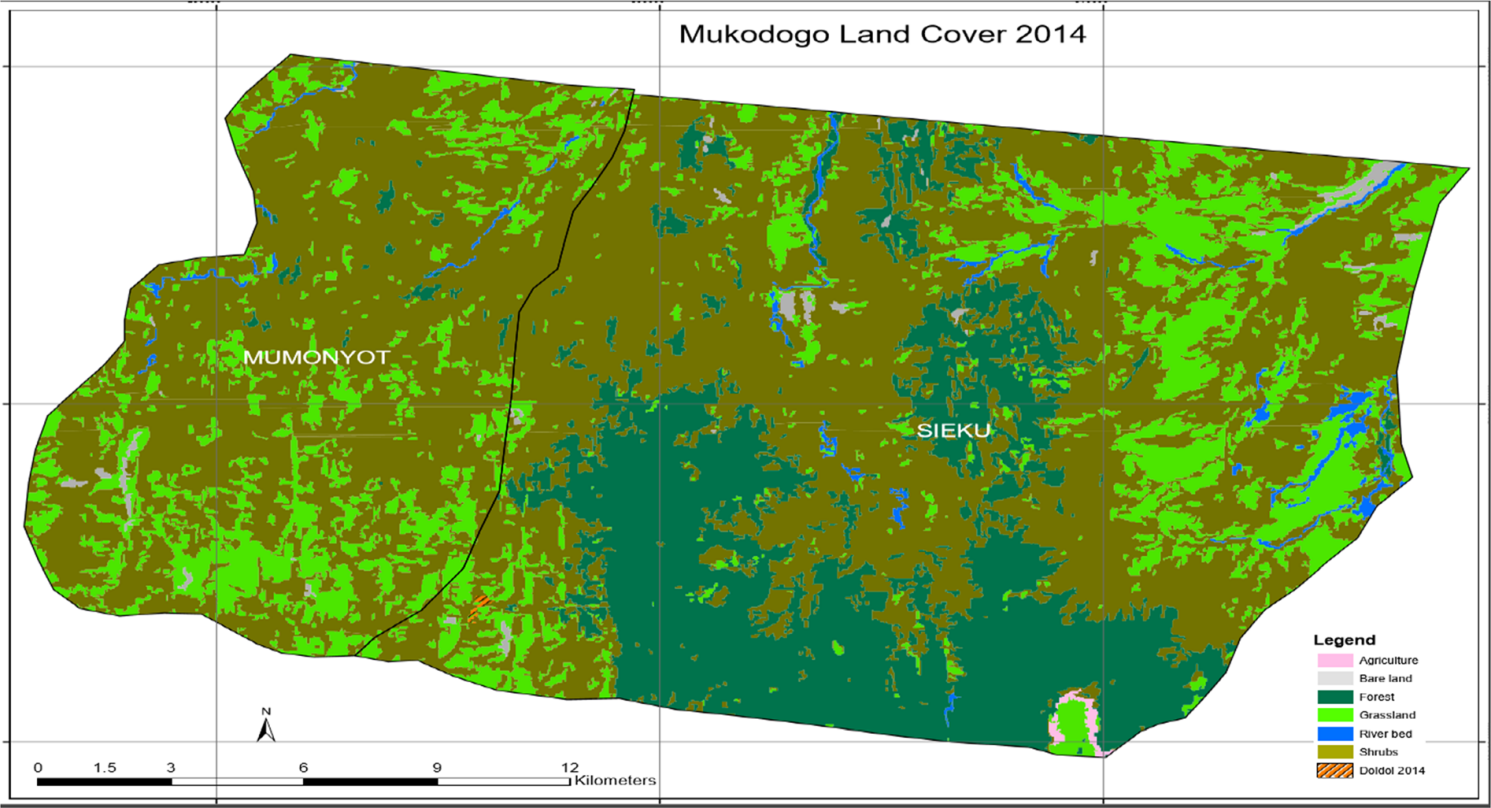
Mukogodo land cover 2014

As indicated in Table 2, between 1995 and 2004, 9213 ha of grassland and 12,919 ha of forest remained unchanged; there was a regeneration of 1584 ha of grassland and a loss of 2406 ha of grassland. In total, there was a loss of 822 ha of grassland. Deforestation counted for 29 ha of loss of forest in this epoch. Agricultural activity had no evidence in that epoch. Plate 7 shows Mukogodo land cover 2014.

**Plate 8:**
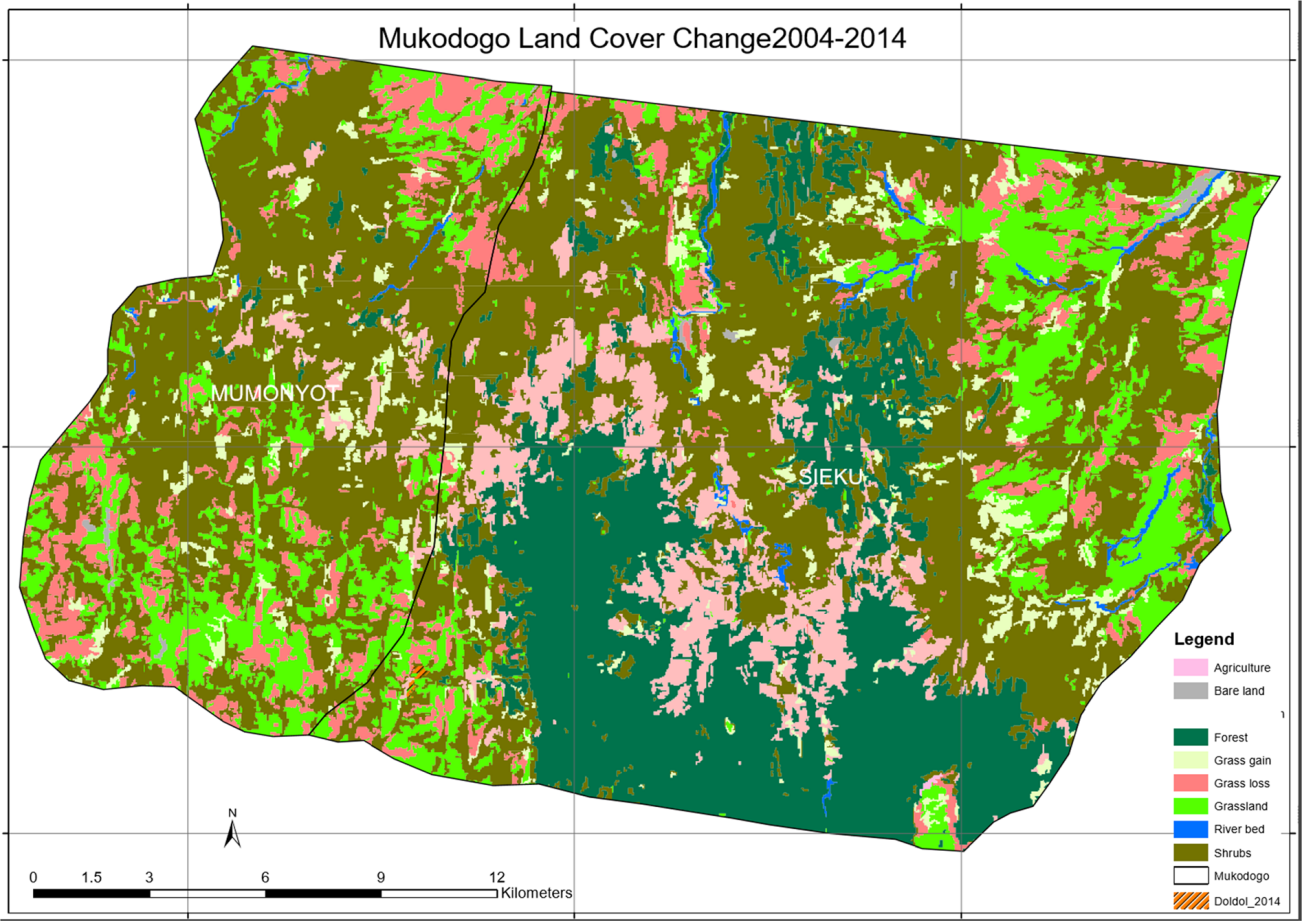
Land cover change 2004–2014

As indicated in Plate 7, the epoch of 2014, agricultural activity increased by more than 600% in this fourth epoch and the area was covered by forest (the green and dark green colour in Plate 6). In this epoch shrub land had been noticed encroaching areas which had grassland. Grassland continued to reduce gradually, of which by 2014, it had reduced by 40% as from 1984. The increase in agricultural activity during this epoch was attributed at Focus group discussion stage to be due to population increase and changing of livelihoods from pastoral to crop farming due to changing climate. Plate 8 shows Mukogodo land cover change 2004 to 2014(overlays map).

As indicated in Plate 8, in the period 2004 to 2014, the grasslands were lost and forested areas remained unchanged. Some areas of grassland regenerated while others lost grass cover giving a net grass loss. Deforestation continued. Table 3 summarises the land use change in 2004 to 2014 as indicated in the Plates 7 and 8.

**Table 3:** 2004–2014 epoch

T1_class_l	2004_LC	2014_LC	Change	Area
14	Grassland	Grassland	Grassland	6875.922483
12	Shrubs	Shrubs	Shrubs	22,858.861236
11	Bare land	Bare land	Bare land	147.327158
14	Grassland	Shrubs	Grass loss	3921.28586
14	River bed	River bed	River bed	351.417255
14	Shrubs	Grassland	Grass gain	1982.861362
9	Forest	Forest	Forest	9661.442734
12	Forest	Shrubs	Deforestation	3304.277771
12	Shrubs	Agriculture	Agriculture	8.82

As indicated in Table 3, between 2004 and 2014, 6876 ha of grassland, 22,859 ha of shrubs and bush-land and 9661 ha of forest remained unchanged. There was however a regeneration of 1983 ha of grassland against a more than double (3921 ha) loss of grassland. Deforestation was also at a record high of 3304 ha. Table 4 summarises the total land use change in 1984 to 2014 as indicated in Plates 2, 3, 4, 5, 6, 7 and 8.

**Table 4:** Land use change in 1984 to 2014

Epoch	Land cover area in hectares						Total land cover
	Agriculture	Bare land	Forest	Grassland	River bed	Shrubs	
1984	9	527	12,732	14,723	292	20,829	49,112
1995	9	309	12,710	11,619	331	24,134	49,112
2004	–	173	12,919	10,797	331	24,893	49,113
2014	61	289	9661	8859	501	29,741	49,112

As indicated in Table 4, in the first three epochs (1984, 1995, 2004), there was very little agricultural activity which increased by more than 600% in the fourth epoch, of which the third epoch (2004) had no agricultural activity at all. However, agriculture covered less than 0.2% of the total land cover. Grassland continued to reduce gradually, of which by 2014, it had reduced by 40% as from 1984. The beneficially class cover was the shrubs and wood land which increased from 20,829 ha in 1984 to 29,741 ha in 2014. An average increase in hectares of shrubs and wood land of 42% in the three decades, with corresponding reduction of forest canopy from 12,732 ha in 1984 to 9661 in 2014 and a decrease of 24% gave the trend of vegetation changes. These hectares include the registered forest of 30,000 ha and the adjacent community conservation areas totalling to about 50,000 ha (C G L. 2017).

**Fig. 2:**
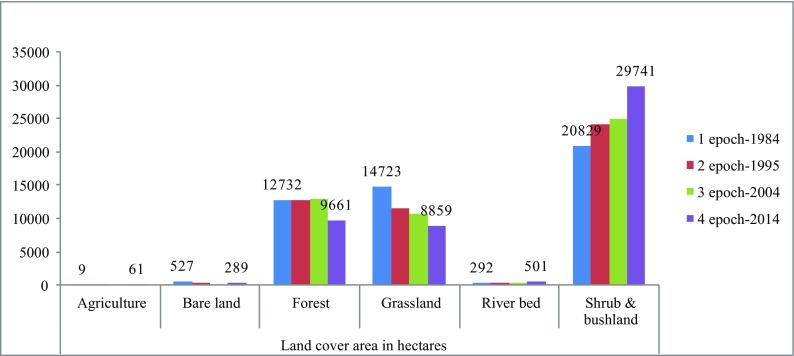
Specific land use change per epoch

This part summarises the results and discussions of determination of the change in land use and management in the last 30 years. Figure 2 shows specific land use change per epoch.

In general as indicated by Fig. 2, the land use types of study area were six, of which three were the main and the other three are the minor. The main three, forest, shrub or bush land and grassland, changed during the last three decades, of which grasslands reduced by 5864 ha (40%), forest by 3071 ha (24%) and shrub and bush land increased by 8912 ha (43%). The other three minor land use types were bare land which had reduced by 238 ha (45%), river bed vegetation increased by 209 ha (72%) and agriculture increased by 52 ha (600%) over the last three decades.

### Opinions of the inhabitants of the ecosystem on the changes

#### Changes in harvesting of forest products

**Fig. 3:**
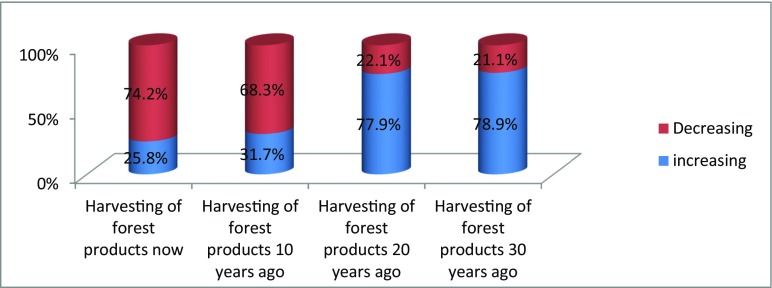
Respondents opinion on changes in harvesting of forest products

Figure 3 shows changes in harvesting of forest products.

Unlike the common understanding that forest resources utilisation increases with increasing human population, the Mukogodo case is different in that the majority of the respondents (78.9%) reported that the forest resource use was more in the last three decades than now and also a similar majority (74.2%) had the same opinion that forest resource utilisation was low compared to the last 30 years as indicated by Fig. 3.

#### Changes in species biodiversity (trees shrubs, herbs, pastures)

**Fig. 4:**
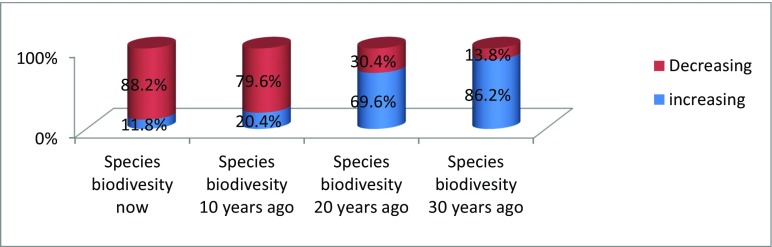
Changes in species biodiversity

Figure 4 shows the changes in species biodiversity.

Like in the case of forest resource utilisation, where the trend of resources utilisation was more in the last three decades than now, the majority of the respondents (86.2%) also indicted that species biodiversity were more in the 30 years ago and decreased progressively to present date as indicated in Fig. 4.

**Fig. 5:**
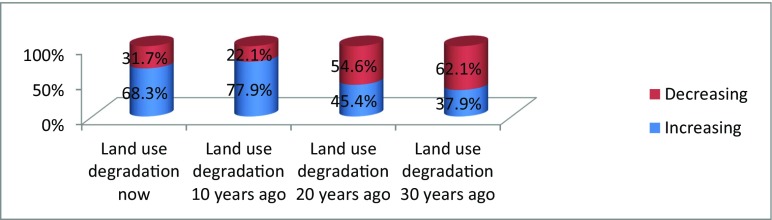
Changes in land use (degradation)

Figure 5 shows changes in land use (degradation).

The majority of the respondents (62.1%) reported that the changes in land use (degradation) had decreased in the last three decades than now, and also, a similar majority (68.3%) had the same opinion that changes in land use (degradation) increased compared to the last 30 years as indicated by Fig. 5.

#### Climatic parameter changes

**Fig. 6:**
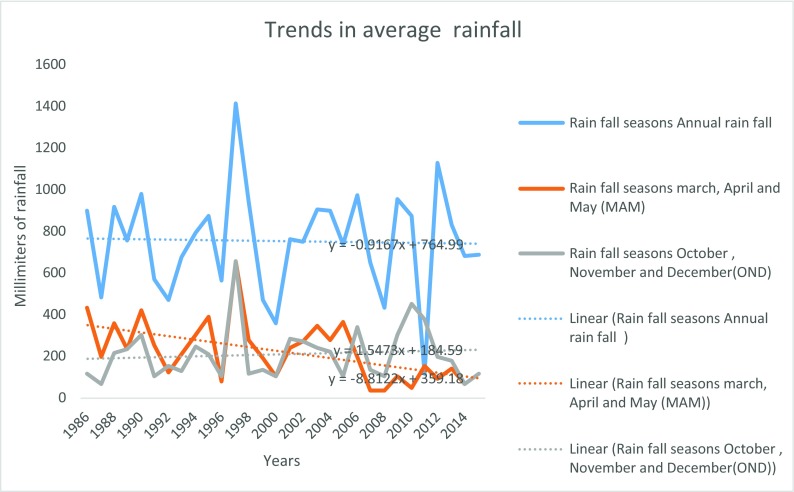
Mukogodo area average annual rainfall trends

Figure 6 shows Mukogodo area average annual rainfall trends.

As indicated in Fig. 6, Mukogodo rainfall trends had different scenarios from other part of Laikipia in that although the annual average rainfall increased in the last three decades, the March, April and May (MAM) rainfall trends decreased progressively, while the October, November and December (OND) increased slightly over the three decades.

### The temperature trends of Laikipia County for the last three decades

**Fig. 7:**
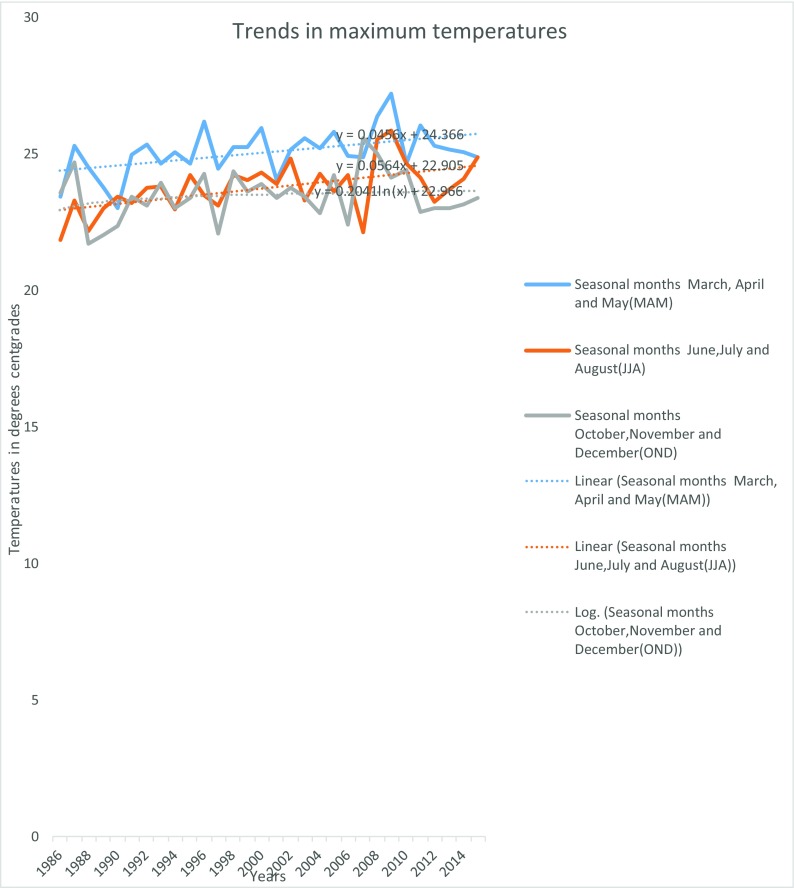
Mukogodo area average annual maximum temperatures

Figure 7 shows Mukogodo average annual maximum temperatures.

**Fig. 8:**
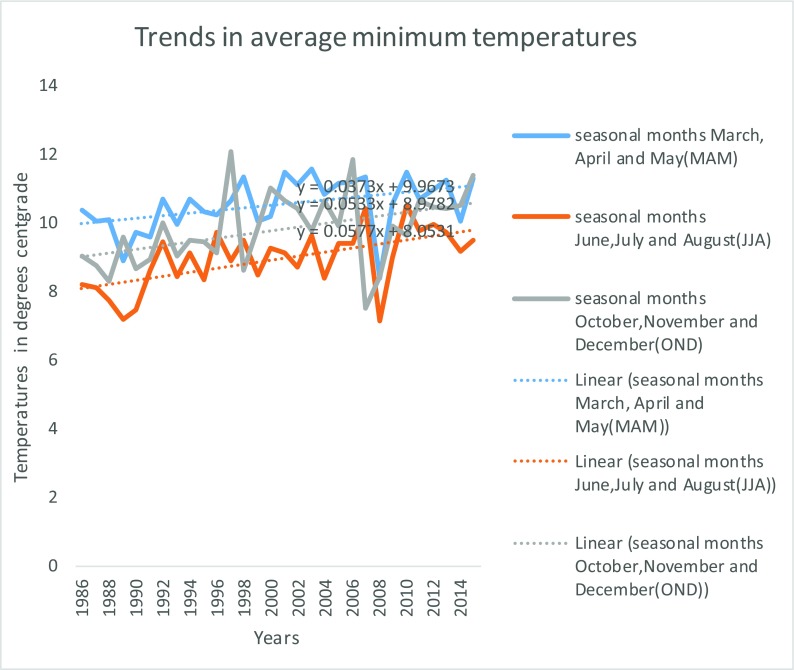
Mukogodo area average annual minimum temperatures

As indicated in Fig. 7, there was an increase in maximum temperatures over the years. Maximum temperatures were higher during the main seasons of MAM and OND but lower in between in the months of June, July and August (JJA). In Mukogodo area, the year 2010 recoded the months with highest temperature and 2007 and 2008 had the lowest temperature, in June, July, August and OND. Generally, there was trend in temperature increase in the whole of three decades with the lowest average annual maximum temperature of 21.7 °C in OND of 1989 and highest average annual maximum temperatures of 27.2 °C in MAM 2009. The maximum temperature increased by 1.43, 2.98 and 0.18 °C during MAM, JJA and OND seasons, respectively, which gave an average increase of 1.5 °C in the period. Figure 8 shows Mukogodo area average annual minimum temperature.

While there was an increase in minimum temperatures over the years as indicated in Fig. 8, minimum temperatures were highest in MAM season followed by OND and lowest between the season in June, July and August. The minimum temperature increased by 0.91, 1.30 and 2.37 °C during MAM, JJA and OND seasons, respectively, which gave an average increase of 1.5 °C in the period.

#### Political influence

**Fig. 9:**
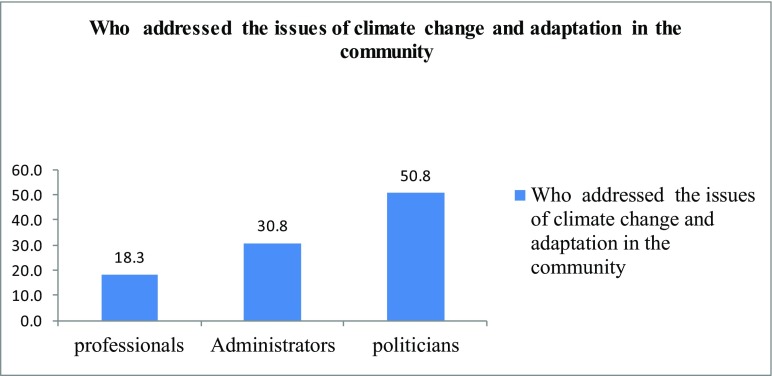
Who addresses the issues of climate change and adaptation in the community

As indicated in Fig. 9, the majority of the respondents (58.5%) in Yaaku community indicated that politicians addressed issues of climate change in the community, with only 18.3 and 30.8% of them that said that climate change issues were addressed by professionals and administrators, respectively. Therefore, the politicians were key in addressing the impacts of climate change although they had not known or understood that the impacts are of climate change but either campaign goodies or development agenda.

## Discussions

The general increase of average rainfall which was not the sum of MAM and OND was due to recoded rainfall in the other months of the year which are not defined seasons of Mukogodo area.

The impacts of climate change led to unreliability of the seasons in which MAM used to be the main long rain season in earlier years, but now, OND has turned to be more reliable in the Mukogodo area although less than normal season during the last three decades. Therefore, the changes have affected the amounts, intensity, distribution, seasonality and reliability of rainfall and thus affected the livelihood of the Yaaku. The rainfall general net decrease of 200 mm was witnessed over the period, of which the most affected were the traditionally known seasons of MAM and OND. The change in rainfall over the study period in Yaaku community of Mukogodo forested ecosystem was attributed to climate change. As evidenced by changes in temperatures and rainfall (Figs. 6, 7 and 8), rainfall decreased by 200 mm and the temperatures increased by 1.5 °C over the period. However, the variability in climate (rainfall and temperatures) had contributed to the change in land use and management of the pastoral forested ecosystem of Mukogodo in which there were decrease of grassland (40%), forest (24%), bare land (45%) and increase of shrub/bush land (43%), riverbed vegetation (72%) and agriculture (600%) cover by the last three decades.

The opinion of the community on the change of land use and management was attributed to climate change and also adaptation strategies applied by the community over time, as evidenced by Niang et al. (2014), Muller et al. (2011) and Sarr (2012). Climate change impacts include shortening or disruption of growing seasons, reductions or increase in the area suitable for agriculture and declines in agricultural yields in many regions of sub-Saharan Africa. In Yaaku community, all these impacts were evidenced and thus mitigation measures were suggested to address the impacts. on harvesting of forest products, species biodiversity (trees, shrubs, herbs, pastures), forest water resources (rivers, springs), wildlife numbers, land use (degradation), livestock keeping (cattle/goats) and bee keeping,.

The phenomenon was contrary to the norm and explained by the community having strong adaptation structure and strategies to impacts of climate change; thus, forest conservation measures were enforced. Kenya Forest Service (KFS) which manages gazetted forest were not managing Mukogodo forest apart from small portion managed by Lekuruki conservancy and proposed Kurikuri conservancy which are community based, but the bigger forested area of the Yaaku community was generally community managed. The same results of increase of forest and shrubby cover are evidenced by remote sensed.

The indication was that although there was increase of forest cover due to reduced use of forest resources, the biodiversity of the vegetation species reduced progressively probably due to effects of climate change. There is a need to have ecological research to document the species composition of the Mukogodo ecosystem and their change over the period. The change of species diversity and degradation of scenery site of caves due to climate change has reduced tourism activity around Mukogodo forest.

As the changes in livelihood system from hunters and gatherers of the Yaaku community to pastoral livelihood, came in with grazing and browsing which lead to ecosystem degradation, in deep discussions with focus group and key informant’s interviews, the degradation was attributed to increase of livestock being grazed in the forested ecosystem.

The adaptations to climate change in the Yaaku Community and in general in the county of Laikipia are addressed by the political class as development issues which are top down approach.

## Conclusions

There are main land use changes in the three decades under study. The main three, forest, shrub or bushland and grassland changed during the last three decades, of which grasslands reduced by 5864 ha (40%), forest by 3071 ha (24%) and shrub and bush land increased by 8912 ha (43%).The other three minor land use types were bare land which had reduced by 238 ha (45%), river bed vegetation increased by 209 ha (72%) and agriculture increased by 52 ha (600%) over the last three decades.

These trends of increase in temperature in the last three decades were attributed to climate change by the Yaaku community. This was inline as evidenced by Thomas et al. (2007) and Songok et al. (2011) that increases in temperature, in sub-Saharan Africa, were expected to cause changes in rainfall intensity due to climate change.

The change of land use and management of the dry pastoral forested ecosystem was due to characteristic of parameters of climate for rainfall decrease by 200 mm and the temperatures increased by 1.5 °C over the period brought about the variability in climate (rainfall and temperatures).

The adaptation strategies applied by the community over time have contributed to change in land use and management as evidenced by (Niang et al. 2014; Muller et al. 2011; Sarr 2012). Climate change impacts include shortening or disruption of growing seasons, reductions or increase in the area suitable for agriculture and declines in agricultural yields in many regions of sub-Saharan Africa.

Our interviews and analysis of the land use and adaptation by the Yaaku community yield evidence of impacts to pastoral land users. These impacts include shorting or disruption of growing seasons and decline in agricultural yield; we also found both reduction and increase in the area suitable for agriculture.

Our interviews and analysis on how to address climate change adaptation issues indicated that political class has a big influence in addressing the climate change adaptations.

## Recommendations

- First, there was need to involve or inform the community through various media, especially through workshops the existence of climate change and its impacts to the ecosystem and community.
- To address changes in land use where shrub or bush land is replacing grazing areas: Controlled bush management and indigenous grass reseeding programme are advocated to restore original grasslands.
- To solicit funds for addressing climate change impacts: The community should use the (CMDRR) report to solicit assistance from national, county government and other stake holders to help address climate change impacts in the pastoral forested ecosystem.
- To address the climate change impact-related issues: increased awareness to government (political class) and other stakeholders on climate change issues for example (allocation of resources and policies of how to utilise those resources) is needed.
